# It starts at the ends: The zebrafish meiotic bouquet is where it all begins

**DOI:** 10.1371/journal.pgen.1007854

**Published:** 2019-01-17

**Authors:** Kellee R. Siegfried

**Affiliations:** Biology Department, University of Massachusetts Boston, Boston, Massachusetts, United States of America; Harvard Medical School, UNITED STATES

Meiosis is a specialized cell-division process in germ cells that ensures proper segregation of a single haploid genome into each gamete from a diploid precursor cell. This process ensures that the embryo receives the correct number of chromosomes upon fertilization. To accomplish this, meiotic germ cells duplicate their genome one time followed by two rounds of cell division (meiosis I and II) to produce haploid gametes. Therefore, there are major modifications that occur compared to mitotic cell divisions to ensure that the two successive meiotic cell divisions faithfully segregate one copy of every chromosome into each gamete [1]. In contrast to mitosis, after DNA replication, the meiotic chromosomes (each consisting of the duplicated sister chromatids) find their counterparts (homologs) and pair. Upon the first meiotic division, these paired homologous chromosomes separate from each other so that each daughter cell ends up with one set of chromosomes. To accomplish this reductional division, homologous chromosomes must find each other and form attachments—or pair, synapse, and form crossovers. Finally, at the second meiotic division, the sister chromatids segregate, resulting in the final outcome of four haploid cells.

**Fig 1:**
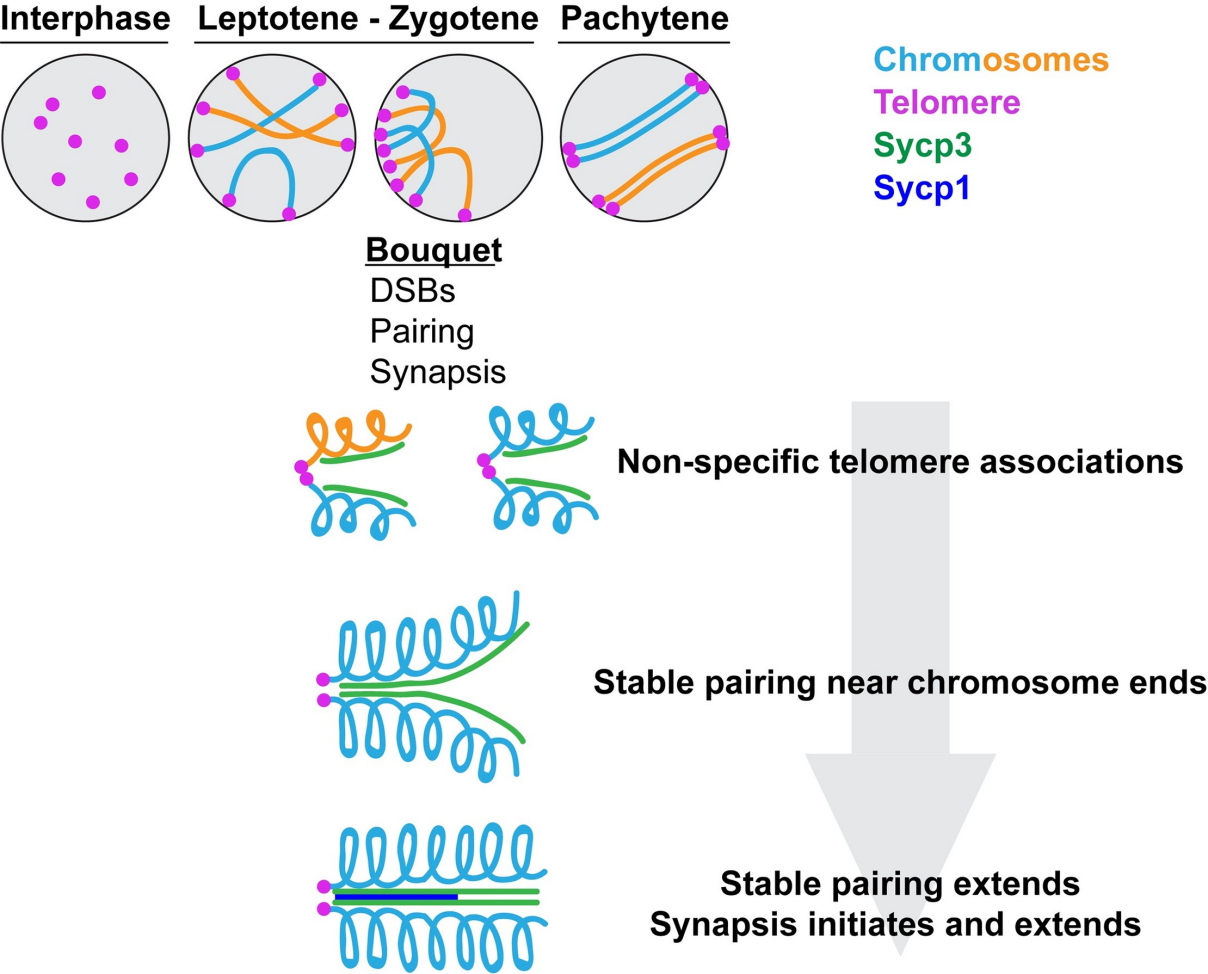
Meiotic DSBs, homologous chromosome pairing, and synapsis are initiated near the chromosome ends in the meiotic bouquet. In the leptotene stage of meiotic prophase, telomeres attach to the nuclear envelope and by early zygotene, move to one region of the nuclear envelope to form the bouquet structure. In the bouquet, DSBs, homologous chromosome pairing, and synapsis between homologs is initiated near the chromosome ends. Telomeres can associate with each other nonspecifically—telomeres from nonhomologous chromosomes are often juxtaposed (nonhomologous chromosomes are represented by different colors). Pairing between homologous chromosome is then seen and initiates close to the telomeres. Synapsis, as visualized by formation of the synaptonemal complex (green lateral element + blue central region), begins in paired regions near telomeres. As pairing extends along the length of the chromosome, synapsis ensues. DSB, double-strand break.

How homologous chromosomes find each other and pair during meiosis I is somewhat of a mystery but seems to involve the telomeres. In many organisms, including vertebrate animals, the telomeres attach to the nuclear envelope at the beginning of meiosis. This attachment is mediated through binding of telomere-associated proteins to a protein complex that spans the nuclear envelope and interacts with the cytoskeleton [2]. The telomeres then move and cluster to one side of the cell forming a structure called the “bouquet” (Fig 1). The movement of the chromosomes during bouquet formation is thought to facilitate pairing of homologous chromosomes. Once homologous chromosomes find each other, they make stable connections, which ensures alignment on the metaphase plate and separation of the two homologs to opposite daughter cells. These stable connections are provided by a proteinaceous structure that forms between homologous meiotic chromosomes, called the synaptonemal complex, and through direct chromosome interactions made through meiotic crossovers [3].

The mechanisms underlying meiotic chromosome segregation have been elucidated through studies on several model organisms, including yeast, fruit flies, nematodes, and mice. The zebrafish has more recently become an additional favorite model system as an intermediate between invertebrates and mammals. Zebrafish research has contributed extensively to many aspects of germ cell biology, including how primordial germ cells are guided to the developing gonad as well as the role of germ cell-specific small RNAs (pi-RNAs) in the germ line, to name a few [4–7]. The zebrafish has many attributes as a model organism, including excellent genetic and genomic tools. In zebrafish, as is true in most animals, gametes in both sexes are continuously replenished throughout the life of the animal. Therefore, the processes of germline stem cell regulation, progenitor cell-divisions, meiosis, and gametogenesis can all be interrogated in a single adult ovary or testis at any given time. Therefore, all stages of both male and female meiosis are easily investigated in the adult zebrafish.

In this issue, Blokhina and colleagues [8] detail pairing and synapsis in zebrafish meiosis. Using super-resolution microscopy, the authors beautifully show that these processes occur much like what has been reported for other model systems, setting the stage for the utilization of this model for in-depth investigations into meiotic mechanisms. The authors demonstrate that telomere-driven bouquet formation is key to the initiation of meiotic pairing. During the early bouquet stage, Blokhina and colleagues found that telomeres associate with one another in a nonhomology-driven fashion (Fig 1). At this time, homologous chromosomes also begin to pair in subtelomeric regions. Therefore, “bunching” together of chromosome ends in the bouquet and general telomere-telomere interactions may serve to bring chromosome ends in close proximity to each other, increasing their chances of finding their homolog and initiating pairing. As pairing is initiated, Synapotemal complex protein 3 (Sycp3), a lateral element component of the synaptonemal complex, accumulates along each chromosome beginning near the ends and extending along the length of the chromosomes. Synapsis then quickly ensues, as visualized by localization of the Sycp1 protein, which is a component of the central region of the synaptonemal complex. Sycp1 localization is initiated at subtelomeric regions, where chromosomes have paired and Sycp3 has been deposited, and continues to follow the extension of Sycp3 deposition along the chromosome. Therefore, pairing initiates near the telomeres in the bouquet. Synapsis quickly follows and extends along the length of the chromosome following paired regions of the chromosomes (Fig 1). DNA double-strand breaks (DSBs), which are a precursor event for crossover formation, are also initially seen near the chromosome ends in the bouquet. Therefore, chromosomal regions near the telomeres and the bouquet configuration are key to pairing and crossover initiation in zebrafish meiosis, similar to what has been found in several other organisms.

To ask how recombination between homologous chromosomes fits into this picture, Blokhina and colleagues generated and characterized zebrafish mutants that were unable to make meiotic DSBs. The SPO11 topoisomerase is a conserved protein that initiates meiotic DSBs [9]. In mice, mutant analysis has demonstrated that the synaptonemal complex can form in the absence of *Spo11* function, but pairing and synapsis are severely impaired [10,11]. In mice, male and female meiosis are affected differently by loss of *Spo11* function. Male *Spo11* mutants display meiotic arrest and apoptosis at the zygotene to mid-pachytene stage. In females, some oocytes persist beyond this stage and a few oocytes mature. However, both sexes are infertile. The ability of female oocytes to continue through oogenesis despite the meiotic defects points to a commonly observed feature of female meiosis in which oocytes seem to have more relaxed checkpoint control relative to males [12].

Bolkhina and colleagues demonstrate that zebrafish *spo11* mutants exhibit similar meiotic defects as those observed in mice. In both male and female mutants, the bouquet forms normally and the assembly of axial elements commences; however, pairing and synapsis are severely impaired. As in mammals, zebrafish mutant males exhibit meiotic arrest, spermatozoa are not present, and males are sterile. By contrast, female mutants produced normal numbers of eggs, yet embryos were generally not viable and died before feeding stages (prior to 5 days post fertilization). This suggests that female meiosis may have even more relaxed checkpoint regulation in zebrafish than in mammals, because egg production is not compromised. Analysis of *mutL homolog 1 (mlh1)* mutant zebrafish, encoding a protein involved in recombination, demonstrates that females with meiotic defects produce a small number of triploid offspring that are viable and develop as sterile males [13]. A similar phenomenon may be happening in the zebrafish *spo11* mutants, because rare viable offspring were produced from mutant females, all of which were male. Overall, the similarities in meiotic processes observed between mammals and zebrafish further validate the zebrafish model as a key resource from which we can gain new insights into the mechanisms underlying meiosis.

The analysis of zebrafish meiotic chromosomes at high resolution is a key advance in our ability to utilize the attributes of this model for studying defects in spermatogenesis and oogenesis. The many similarities to mammalian meiosis reported by Bolkhina and colleagues sets the stage for in-depth analysis of meiotic chromosome interactions and utilization of other mutant resources to tease apart mechanistic gaps in our knowledge of this process. This experimental system is primed to address unanswered questions, such as how homologous chromosome find each other and pair in a homology-directed fashion, as well as the role that telomere-directed bouquet formation plays in this process.
